# ANGPTL4, a direct target of hsa-miR-133a-3p, accelerates lung adenocarcinoma lipid metabolism, proliferation and invasion

**DOI:** 10.18632/aging.205313

**Published:** 2023-12-29

**Authors:** Qihao Hu, Shi Chen, Yukun Li, Teng Hu, Jianpeng Hu, Cheng Wang, Fei Yang, Xiang Yang, Feng Zhou, Zhengdong Liu, Wei Xu, Ji Zhang

**Affiliations:** 1 Department of Thoracic Surgery, The First People’s Hospital of Changde City, Changde, Hunan, China; 2 Department of Assisted Reproductive Centre, Zhuzhou Central Hospital, Xiangya Hospital Zhuzhou Central South University, Central South University, Zhuzhou, Hunan, China; 3 Department of Pathology, The First People’s Hospital of Changde City, Changde, Hunan, China

**Keywords:** ANGPTL4, lung adenocarcinoma, hsa-miR-133a-3p, lipid metabolism reprogramming, bioinformatics

## Abstract

Background: Globally, lung adenocarcinoma (LUAD) is the most common type of lung cancer. The secreted protein angiopoietin-like 4 (ANGPTL4) has been implicated in a number of physiological and pathological processes, including angiogenesis and lipid metabolism. But the role of ANGPTL4 in LUAD remains unknown.

Methods: The expression of ANGPTL4 and miR-133a-3p was confirmed by public database analysis. Xenograft model, MTT, Clone formation and EdU analysis were used to confirm the effects of miR-133a-3p/ANGPTL4 on LUAD cell proliferation and growth. Wound healing and Transwell analysis were used to elucidate the role of miR-133a-3p/ANGPTL4 in LUAD cell migration and invasion. Oil red O staining was used to confirm ANGPTL4 in LUAD lipids production. Dual-luciferase reporter gene analysis was used to demonstrate miR-133a-3p could directly bind ANGPTL4 3′-UTR. WB and PCR were used to confirm the protein expression of ANGPTL4.

Results: ANGPTL4 was significantly increased in LUAD samples, which could promote LUAD cell proliferation, migration, invasion, growth and lipid production. miR-133a-3p could directly bind to ANGPTL4 mRNA, and repress the expression ANGPTL4, resulting in suppressing LUAD proliferation and metastasis.

Conclusion: In conclusion, miR-133a-3p/ANGPTL4 axis might be a potential biomarker and therapeutic target for LUAD patients.

## INTRODUCTION

Lung adenocarcinoma (LUAD), the most common form of lung cancer, is the leading cause of cancer-related deaths worldwide [1]. Despite significant advancements in cancer treatment, the prognosis for LUAD patients remains poor due to the heterogeneity and complexity of the disease [2]. LUAD is characterized by complex genomic alterations, which contribute to disease initiation, progression, and metastasis [3]. Therefore, there is an urgent need for further research into the cellular and physiopathologic mechanisms underlying lung adenocarcinoma to develop more effective treatments.

ANGPTL4 is a secreted protein that has been implicated in multiple physiopathologic processes, including lipid metabolism, and cancer progression [4]. Through its roles in these processes, ANGPTL4 has garnered significant attention as a potential therapeutic target [5]. Specifically, ANGPTL4 has been found to play a role in the regulation of vascular permeability and angiogenesis, which has implications for the treatment of various vascular disorders, such as diabetic retinopathy and cancer [6]. Furthermore, ANGPTL4 has been shown to have profound effects on lipid metabolism, affecting both uptake and storage, and has been associated with obesity and metabolic disorders [7]. Most recently, ANGPTL4 has been implicated in cancer cell migration, invasion, and metastasis [8]. As such, ANGPTL4 represents an exciting therapeutic target in the fight against cancer [9]. Nevertheless, the specific functions of ANGPTL4 and the exact regulatory mechanisms affecting its expression in LUAD are yet to be fully understood.

MicroRNAs (miRNAs), a class of small non-coding RNAs, is involved in post-transcriptional regulation by suppressing target gene expression [10]. Among them, miR-133a-3p is involved in various aspects of cancer development and progression [11–13]. Dysregulation of miR-133a-3p has been found to promote cell proliferation, migration, invasion, and angiogenesis in several types of cancer, including lung cancer [14, 15]. Furthermore, miR-133a-3p has also been identified as an important diagnostic and prognostic biomarker in lung cancer, with its expression levels correlating with the clinical stage and outcome of the disease [16].

In this study, we found that ANGPTL4 is ectopic expressed in LUAD and that its upregulation is correlated with an unfavorable prognosis. Silencing ANGPTL4 inhibited the viability of LUAD cells. Moreover, miR-133a-3p negatively regulated ANGPTL4 through an interaction with its 3′-UTR. According to our findings, miR-133a-3p/ANGPTL4 might be utilized as a potential therapy in LUAD.

## METHODS

### Bioinformatic analysis

The expression analysis for 522 cases LUAD patient samples and 59 cases paracancerous tissue samples were based on TCGA database LUAD dataset (https://portal.gdc.cancer.gov/) [17]. GSEA and GO analysis was based on LinkedOmics database (http://www.linkedomics.org/login.php) [18]. miRNA analysis was based on TargetScan database (https://www.targetscan.org/vert_80/) [19]. The overall survival of 522 cases of LUAD patient samples were also based on TCGA database LUAD dataset (https://portal.gdc.cancer.gov/) [17].

### Cell culture and transfection

The LUAD cell lines (A549 and H1975) were obtained from the American Type Culture Collection (USA) and cultured in DMEM with 10% fetal bovine serum (FBS), 100 IU/mL penicillin, and 10 μg/mL streptomycin. We purchased ANGPTL4 shRNA, negative control vectors, ANGPTL4 OE, miR-133a-3p mimics, and miR-133a-3p inhibitors from HonorGene (Changsha, China) and used Lipofectamine 3000 (Thermo Fisher Scientific, USA) for transfecting the cells.

### qRT-PCR

For specific methods, please refer to our previous publication [20]. The primer sequences were as follows: miR-133a-3p F: 5′-CTTTAACCATTCTAGCTTTTCCAGGTA-3′ R: 5′-GACTTCGGCTGTGGACAAGATTAG-3′ U6 F: 5′-CTCGCTTCGGCAGCACACA-3′ R: 5′-AACGCTTCACGAATTTGCGT-3′ ANGPTL4 F: 5′-TCCTGGACCACAAGCACCTAGAC-3′ R: 3′-CGGTTGAAGTCCACTGAGCCATC-5′ β-actin F: 5′-GGGACCTGACTGACTACCTC-3′ F: 5′-TCATACTCCTGCTTGCTGAT-3′.

### Western blot

For specific methods, please refer to our previous publication [20]. The primary antibodies are ANGPTL4 (ab115798, Abcam, UK) and FASN (ab128870, Abcam).

### IHC staining

For specific methods of IHC staining, please refer to our previous study [20]. The primary antibody for IHC were as follows: PCNA (ab265609, Abcam), E-cadherin (ab227639, Abcam), N-cadherin (ab76011, Abcam) and ANGPTL4 (ab196746, Abcam).

### Proliferation analysis

For the MTT assay, 5000 cells were cultured in 96-well plates. After incubation, 20 μl of MTT solution (5 mg/ml, Sigma–Aldrich, USA) was added to each well, and the plates were further incubated for 4 hours at 37°C with 5% CO_2_. Next, 150 μl of DMSO was used to dissolve the precipitates.

For the colony formation assay, cells in the logarithmic growth phase were digested with 0.25% trypsin, suspended in DMEM culture medium containing 10% FBS, and the cell suspension was serially diluted. Then, 50 cells from each group were seeded into dishes containing 2 mL of preheated culture medium and incubated at 37°C with 5% CO_2_ for 14 days. The cells were gently rotated to disperse them evenly during incubation.

For the EdU assay, the kit instructions provided by RiboBio (Guangzhou, China) were followed.

### Migration and invasion analysis

For the wound healing study, 5 × 10^5^ cells were placed in 6-well plates, and the surface was scratched using a pipette tip. After washing the cells with DMEM, they were cultured at 37°C for 24 hours. Photographs were taken at 0 and 24 hours to evaluate the healing process.

For the Transwell assay, 2.5 × 10^5^ cells were seeded in a 24-well Transwell chamber (Costar, USA) with Matrigel for the invasion assay. The cells were cultured in DMEM with a 10% FBS concentration for 16 hours at 37°C with 5% CO_2_. The invading cells on the filter’s lower surface were stained with crystal violet.

### Dual-luciferase reporter gene

In A549 cells, luciferase assays were performed. Genes from wild-type ANGPTL4 and mutant controls were integrated into the psiCheck2 Luciferase vector (Promega Corporation, USA). We purchased the psiCHECK2 vector (cat. no. C8021) from Promega. We synthesized the miR-133a-3p mimics, negative control samples (NCs), and miR-133a-3p inhibitor and NC inhibitor at Guangzhou RiboBio Co., Ltd. (Guangzhou, China), which were transfected with Lipofectamine^®^ 2000 (Invitrogen; Thermo Fisher Scientific). Firefly and Renilla luciferase activities were measured 48 h after transfection using the dual-luciferase assay kit (Promega Corporation). In the next step, cells were collected and lysed. On a Luminometer TD20/20 detector (E5311; Promega Corporation), luciferase activity was measured using the Dual Luciferase Reporter Assay System Kit (Promega Corporation). Finally, the relative activity of Firefly/Renilla luciferase was measured.

### Xenograft model assay

The Vector and ANGPTL4 KD A549 cells were subcutaneously injected into adults with athymic BALB/c nude mice (4 weeks old). Each ten days, tumor volumes (cm^3^) were measured. The volume = width^2^ × length × 0.5. As a next step, the xenografts were measured at 50 days. In the last step, the xenografts were dehydrated and sectioned in order to be stained.

### Statistical analysis

The statistical analyses were conducted using R language (Version 3.6). All statistical tests were two-tailed, and a significance level of *P* < 0.05 was deemed statistically significant.

### Data availability statement

The datasets presented in this study can be found in online repositories. The names of the repository/repositories and accession number(s) can be found in the Article/Supplementary Materials.

## RESULTS

### The expression and potential function of ANGPTL4 in LUAD

**Figure 1:**
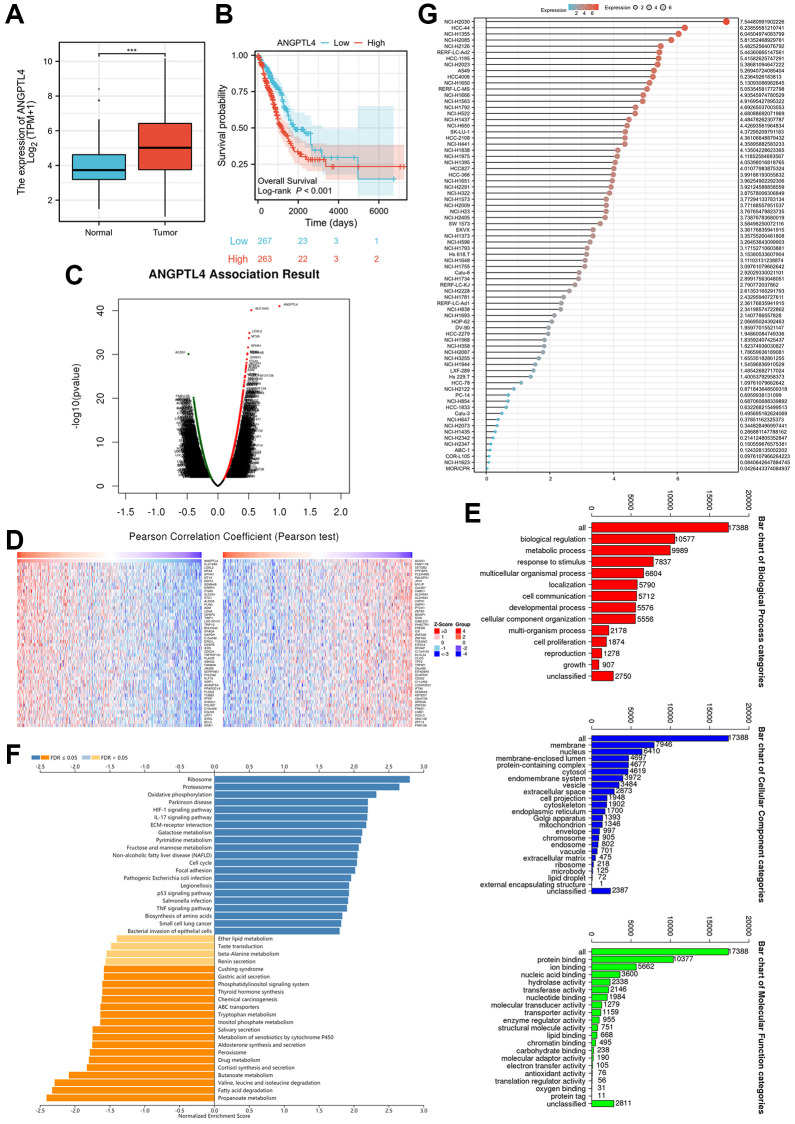
**The expression and potential function of ANGPTL4 in LUAD.** (**A**) The expression of ANAGPTL4 in LUAD patients based on TCGA database. (**B**) The overall survival of ANGPTL4 for LUAD patients. (**C**) The correlated gene of ANGPTL4 in LUAD patients by heat map. (**D**) The correlated gene of ANGPTL4 in LUAD patients by volcanic plot. (**E**) The GO enrichment analysis for these gene. (**F**) The GSEA assay for these gene. (**G**) The expression of ANGPTL4 in LUAD cell lines based on CCLE database. ^***^P < 0.001 indicates statistical significance compared with the control.

Firstly, we utilized the TCGA database LUAD dataset to confirm the expression of ANGPTL4 in LUAD patients, which indicated that ANGPTL4 level was obviously increased in LUAD samples (Figure 1A). Moreover, ANGPTL4 expression was also obviously and positively correlated with poor prognosis in LUAD patients (Figure 1B). LinkedOmics (http://www.linkedomics.org/login.php) was used to access the correlated gene dataset for ANGPTL4 mRNA in LUAD patients (Figure 1C, 1D). GO enrichment assay showed that these gene were enriched in biological regulation, metabolic process, and response to stimulus for BP term, membrane, nucleus, membrane-enclosed lumen for CC term, protein binding, ion binding, and nucleic acid binding for MF term (Figure 1E). GSEA assay indicated that these genes were enriched in ribosome, proteasome, oxidative phosphorylation, ether lipid metabolism and fatty acid degradation (Figure 1F). We used CCLE database to further confirm the ANGPTL4 in multiple LUAD cell lines (Figure 1G). Based on this result, we selected A549 and H1975 as subjects for further studies.

### ANGPTL4 knockdown impeded proliferation, lipids accumulation and invasion in LUAD cell

**Figure 2:**
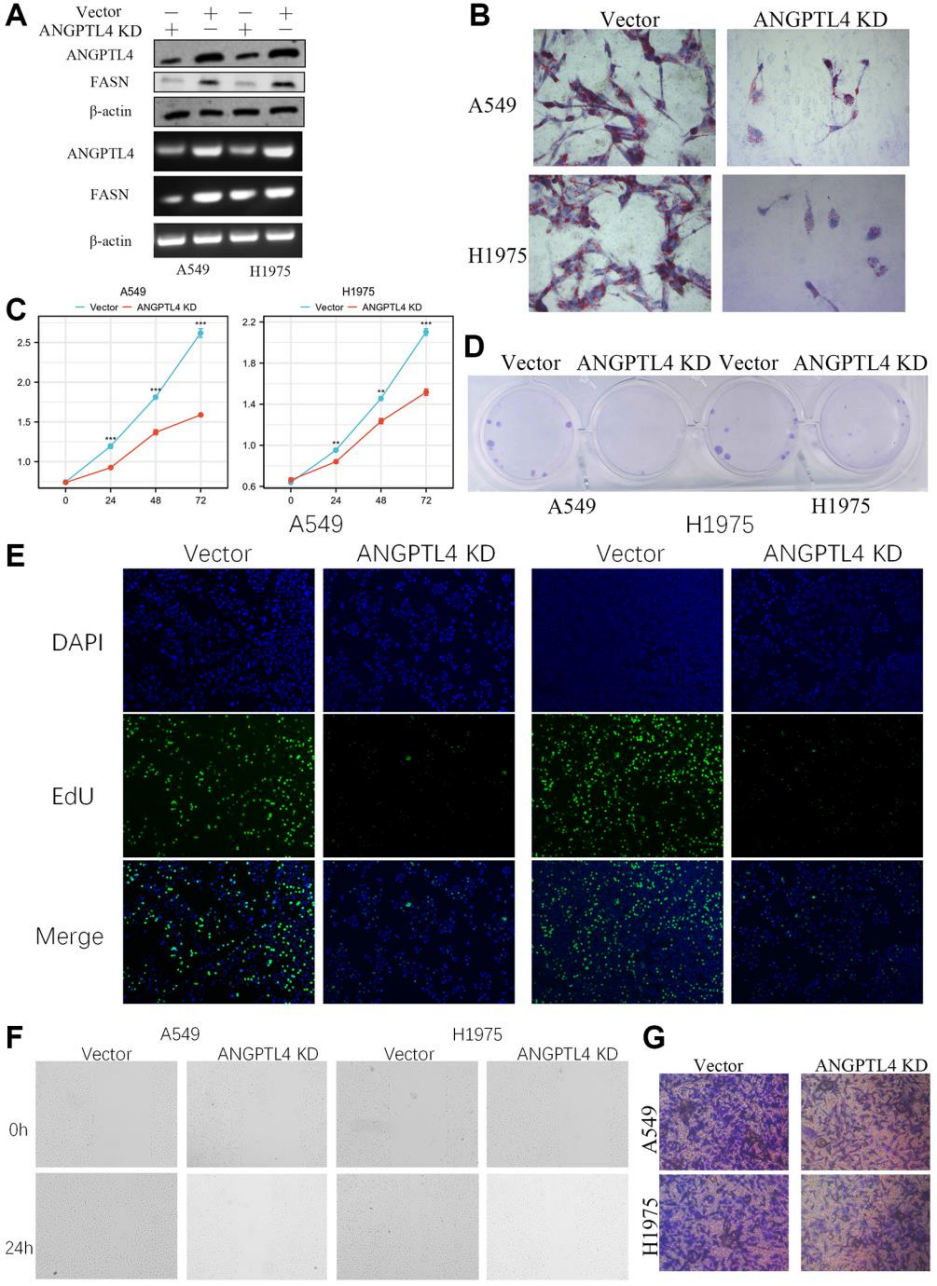
**The effect of ANGPTL4 on LUAD cell proliferation, lipids production, migration and invasion.** (**A**) The expression of ANGPTL4 and FASN in ANGPTL4 KD A549 and H1975 cell by WB and PCR. (**B**) The effect of ANGPTL4 KD on lipids metabolism by oil red O. MTT analysis (**C**), Clone formation assay (**D**), and EdU staining (**E**) were performed to confirm the effect of ANGPTL4 KD on cell proliferation. Wound healing assay (**F**) and Transwell invasion assay (**G**) were performed to confirm the effect of ANGPTL4 KD on cell migration and invasion. ^**^P < 0.01, ^***^P < 0.001 indicates statistical significance compared with the control.

Next, we constructed ANGPTL4 knockdown A549 and H1975 cell (Figure 2A). Oil red O staining showed that ANGPTL4 knockdown could significantly downregulate lipids production in A549 and H1975 cell (Figure 2B). MTT analysis showed that ANGPTL4 knockdown could significantly repress LUAD cell viability (Figure 2C). The clone formation assay showed that the ANGPTL4 inhibition could obviously impeded the clone formation ability in A549 and H1975 cell (Figure 2D). EdU assay showed that the DNA replication level was remarkedly decreased after ANGPTL4 knockdown in LUAD cell (Figure 2E). Wound healing analysis showed that ANGPTL4 inhibition could significantly downregulate the migration ability in A549 and H1975 cell (Figure 2F). Transwell invasion assay showed that ANGPTL4 knockdown could obviously decrease the invasion ability for LUAD cell (Figure 2G).

### Silencing ANGPTL4 inhibits LUAD growth *in vivo*

**Figure 3:**
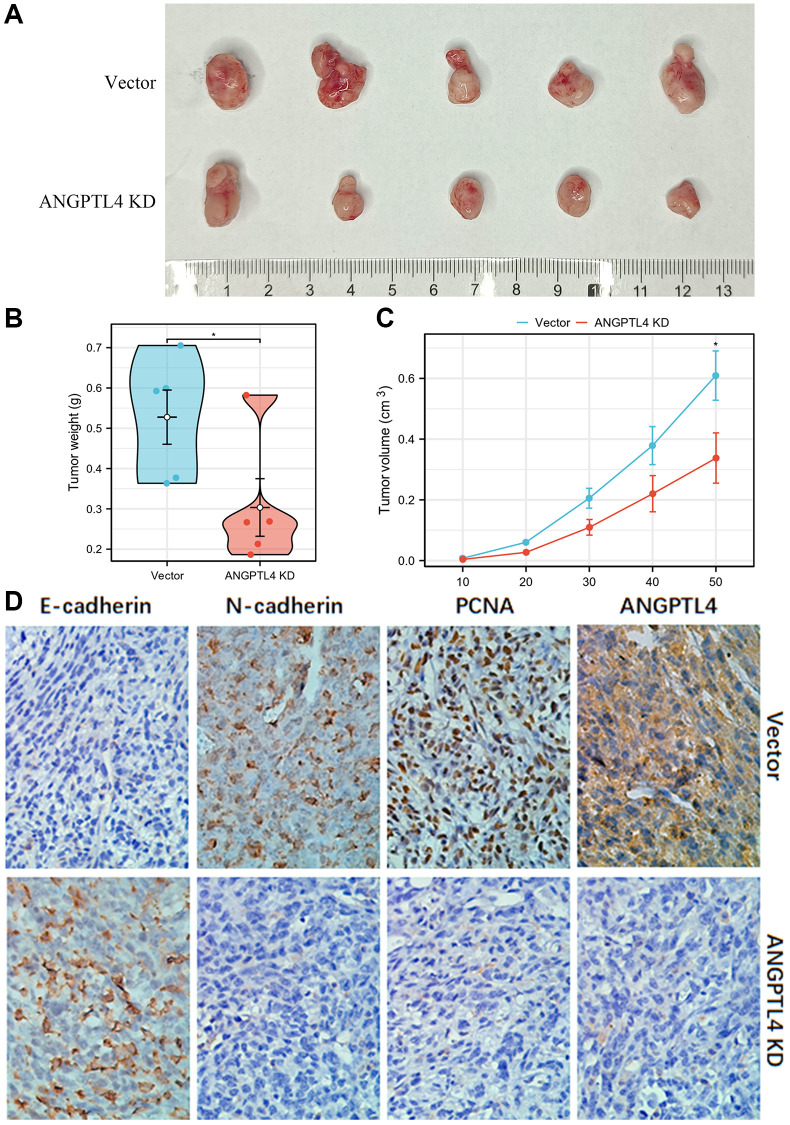
**The effect of ANGPTL4 on LUAD cell growth *in vivo*.** (**A**) The effects of ANGPTL4 shRNA lentivirus plasmid transfection or empty vector lentivirus plasmid transfection on A549 growth *in vivo*. The tumor weight (**B**) and volume (**C**) of the xenografts in ANGPTL4 KD group and vector group. (**D**) IHC staining for E-cadherin, N-cadherin, PCNA and ANGPTL4 in these xenografts for confirming the effects of ANGPTL4 on LUAD EMT cascade and proliferation. ^*^P < 0.05 indicates statistical significance compared with the control.

For confirming the function of ANGPTL4 *in vivo*, we used stable ANGPTL4 knockdown or vector A549 cell to inject into nude mice for constructing xenograft model. We found silencing ANGPTL4 significantly attenuated the LUAD growth, with smaller size and less weight of xenografts in ANGPTL4 knockdown group (Figure 3A–3C). IHC staining suggested that N-cadherin and PCNA expression was obviously decreased in ANGPTL4 KD group, but the expression of E-cadherin was obviously increased in ANGPTL4 KD group (Figure 3D).

### ANGPTL4 is negatively mediated by miR-133a-3p

**Figure 4:**
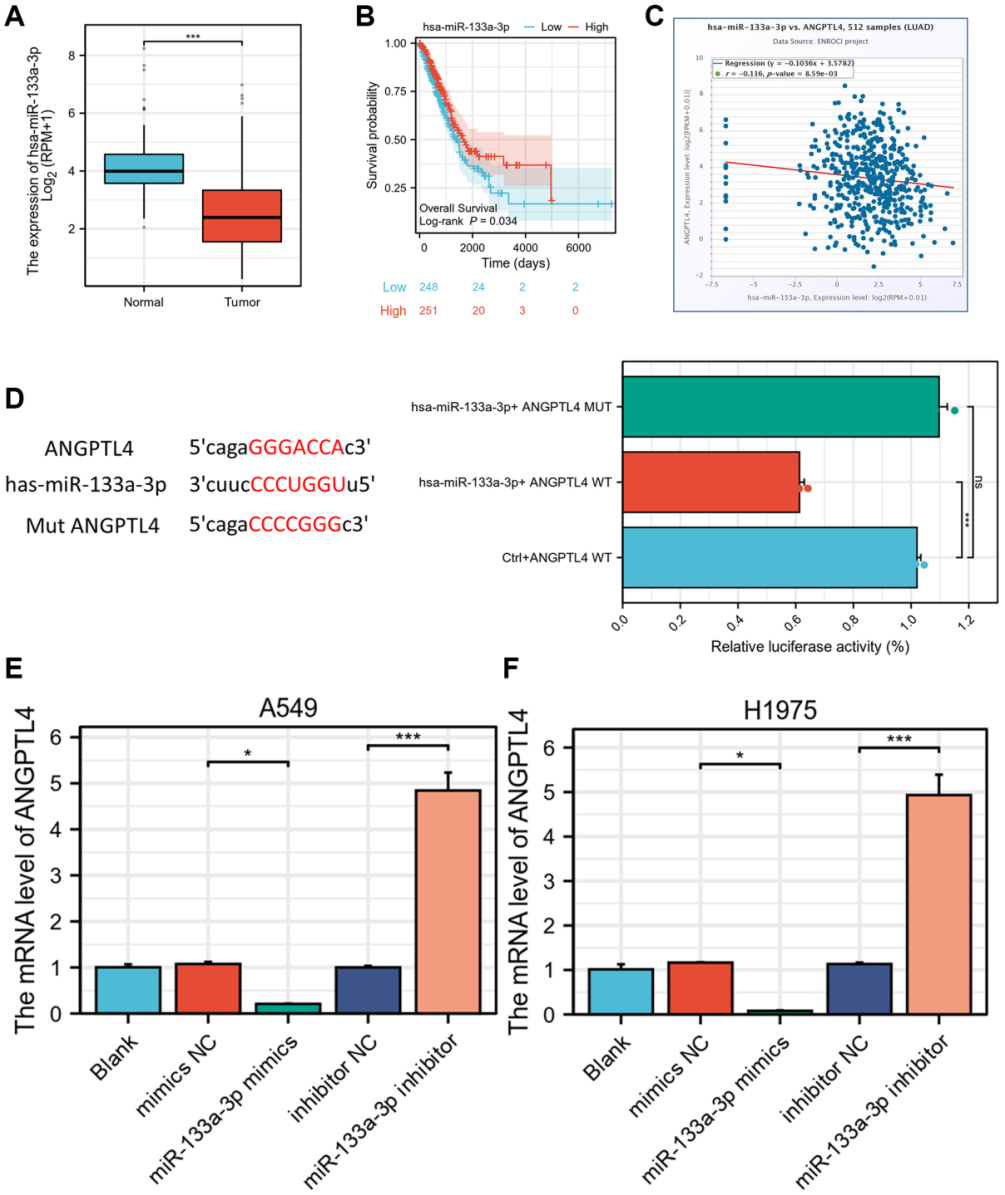
**ANGPTL4 is a direct target of miR-133a-3p.** (**A**) The expression of miR-133a-3p in LUAD patients based on TCGA database. (**B**) The overall survival between high or low level of miR-133a-3p LUAD patients. (**C**) The correlation analysis between ANGPTL4 and miR-133a-3p in LUAD patients. (**D**) Luciferase reporter analysis for WT or MUT 3′-UTR of ANGPTL4 in A549 cell. The qRT-PCR analysis for the effect of miR-133a-3p on ANGPTL4 expression in A549 cell (**E**) and H1975 cell (**F**). ^*^P < 0.05 indicates statistical significance compared with the control. ^*^P < 0.05 and ^***^P < 0.001 indicates statistical significance compared with the control.

To elucidate how ANGPTL4 high expression is in LUAD, we used TargetScan database (https://www.targetscan.org/vert_80/) to seek for the miRNAs targeting ANGPTL4. We found miR-133a-3p has complementary sequences to binding against 3′-UTR of ANGPTL4 (Supplementary Figure 1). There is evidence that miR-133a-3p maybe a TSG in multiple cancer types, including LUAD [13], oesophageal cancer [12], prostate cancer [21], and gastric cancer [22]. We found the level of miR-133a-3p was significantly downregulated in LUAD samples based on TCGA database LUAD dataset (Figure 4A). Moreover, miR-133a-3p was negatively associated with poor prognosis (Figure 4B). The miR-133a-3p was significantly and negatively correlated with ANGPTL4 expression based on starBase database in LUAD patients (Figure 4C). We used luciferase reporter assay to confirm the directly regulation between ANGPTL4 and miR-133a-3p in A549 cell. The luciferase activity was significantly repressed in ANGPTL4 WT plus miR-133a-3p group compared to ANGPTL4 MUT plus miR-133a-3p group (Figure 4D). The qRT-PCR showed that miR-133a-3p mimics could decrease ANGPTL4 mRNA, and miR-133a-3p inhibitor could increase ANGPTL4 mRNA in A549 and H1975 cell (Figure 4E, 4F).

### The miR-133a-3p impeded the proliferation and invasion by targeting ANGPTL4 in LUAD cell

**Figure 5:**
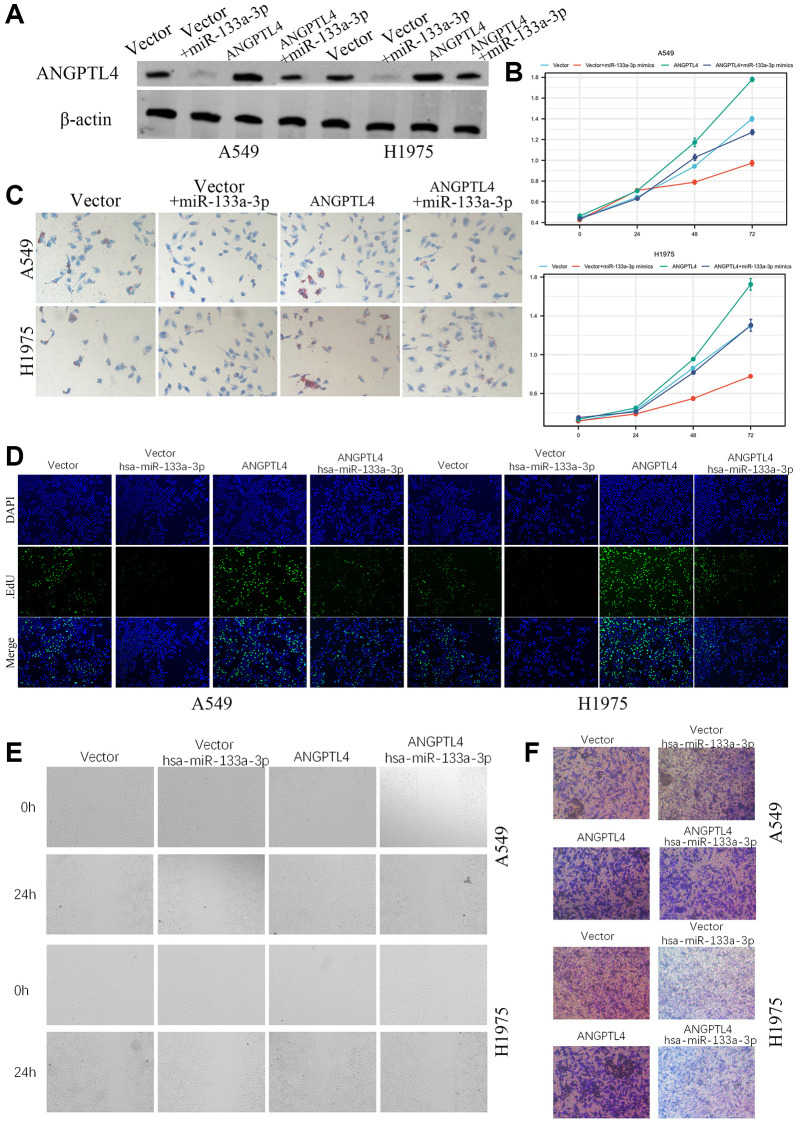
**The role of miR-133a-3p/ANGPTL4 axis in LUAD cell proliferation and invasion.** (**A**) The ANGPTL4 protein expression was confirmed by Western blot. (**B**) The effect of miR-133a-3p/ANGPTL4 axis on LUAD cell proliferation by MTT. (**C**) The effect of ANGPTL4 KD on lipids metabolism by oil red O. (**D**) LUAD proliferation ability for miR-133a-3p/ANGPTL4 axis was showed by EdU analysis. The effect of miR-133a-3p/ANGPTL4 axis on LUAD cell migration by wound healing (**E**) and invasion by Transwell analysis (**F**).

For further confirming miR-133a-3p/ANGPTL4 axis in LUAD development and progression, A549 and H1975 were transfected with vector, vector plus miR-133a-3p mimics, ANGPTL4, ANGPTL4 plus miR-133a-3p mimics (Figure 5A). MTT analysis showed that miR-133a-3p mimics could significantly repress cell proliferation ability, but ANGPTL4 overexpression could amplify cell proliferation ability (Figure 5B). Oil red O showed that ANGPTL4 could significantly increase lipids level, which could be reversed by miR-133a-3p (Figure 5C). EdU analysis showed that the DNA replication level was also upregulation in ANGPTL4 overexpression group, downregulation in miR-133a-3p mimics group and rescued in ANGPTL4 plus miR-133a-3p mimics group in A549 and H1975 cell (Figure 5D). The migration ability was significantly increased in ANGPTL4 group, but decreased in miR-133a-3p group, and rescued in ANGPTL4 plus miR-133a-3p mimics group in A549 and H1975 cell by wound healing assay (Figure 5E). Transwell invasion assay found that the invasion ability of A549 and H1975 cell was significantly decreased by miR-133a-3p and rescued by ANGPTL4 overexpression (Figure 5F).

## DISCUSSION

ANGPTL4 play a key role in a variety of physiological and pathological nonmetabolic and metabolic conditions, such as angiogenesis, tumorigenesis, glucose homeostasis, lipid metabolism, energy homeostasis, immune infiltration, inflammation, and redox regulation [8]. There is evidence that ANGPTL4 is ectopically expressed in certain malignant tumor tissues, including ovarian cancer [23], cervical cancer [24], colorectal cancer [25], pancreatic cancer [26] and papillary thyroid cancer [27]. In this study, we found ANGPTL4 was significantly upregulated in LUAD patients, and correlated with poor prognosis.

Moreover, our results indicated that ANGPTL4 knockdown inhibited LUAD cell proliferation, lipids production and invasion *in vitro*, and attenuated LUAD growth *in vivo*. Previous study indicated that ANGPTL4 could bind to LPL and inhibit its biological activity to decrease lipids metabolism [28]. The mRNA level of LPL was significantly decreased in LUAD patients [29]. Moreover, LPL might be an excellent candidate target for cancer prevention or therapy in multiple cancer types [30]. We also found ANGPTL4 knockdown could significantly reduce the proliferation, migration and invasion ability in LUAD cell. Other study found that ANGPTL4 could regulate glutamine metabolism and fatty acid oxidation in lung cancer cells [31]. Shen et al. found that oleic acid could promoted colon cancer metastasis by driving ANGPTL4 [32]. Fang et al. indicated that he expression of ANGPTL4 regulated pyroptosis and apoptosis in LUAD cells by driving NLRP3/ASC/Caspase 8 pathway, leading to gefitinib resistance in those cells [33]. Zhu et al. found that ANGPTL4 could promote epithelial-mesenchymal transition cascade via ERK Pathway in lung cancer [34]. These results both indicated that ANGPTL4 might be a key role in LUAD carcinogenesis, which was still a need for further investigation.

We further found miR-133a-3p could remarkedly repressed ANGPTL4 mRNA expression via binding to its 3′-UTR, and the levels of miR-133a-3p was significantly decreased in LUAD patients. Li et al. found that miR-133a-3p could attenuate gefitinib resistance by targeting SPAG5 in LUAD [13]. Xu and his colleagues found that miR-133a-3p could suppress the LUAD malignant behavior via targeting ERBB2 [14]. Pan and his colleagues found that miR-133a-3p could interact with PURB to mediate MAPK and PI3K/Akt pathway in LUAD cell [35]. In this study, we found miR-133a-3p was correlated with favourable prognosis for LUAD patients, and negatively correlated with the ANGPTL4 mRNA expression to impede LUAD cell proliferation, migration and invasion. Taken together, these results both indicated that miR-133a-3p has multiple downstream targets involved in LUAD progression due to its complex regulation mechanism.

This study has several limitations that should be addressed in the future. Firstly, the mechanism and signaling pathway through which ANGPTL4 regulates lung cancer cell proliferation and invasion remain unknown. Secondly, it needs to be clarified whether miR-133a-3p regulates ANGPTL4 exclusively through negative regulation or whether it also controls other miRNAs. Furthermore, the potential value of ANGPTL4 as a prognosis biomarker should be further investigated.

**Figure 6:**
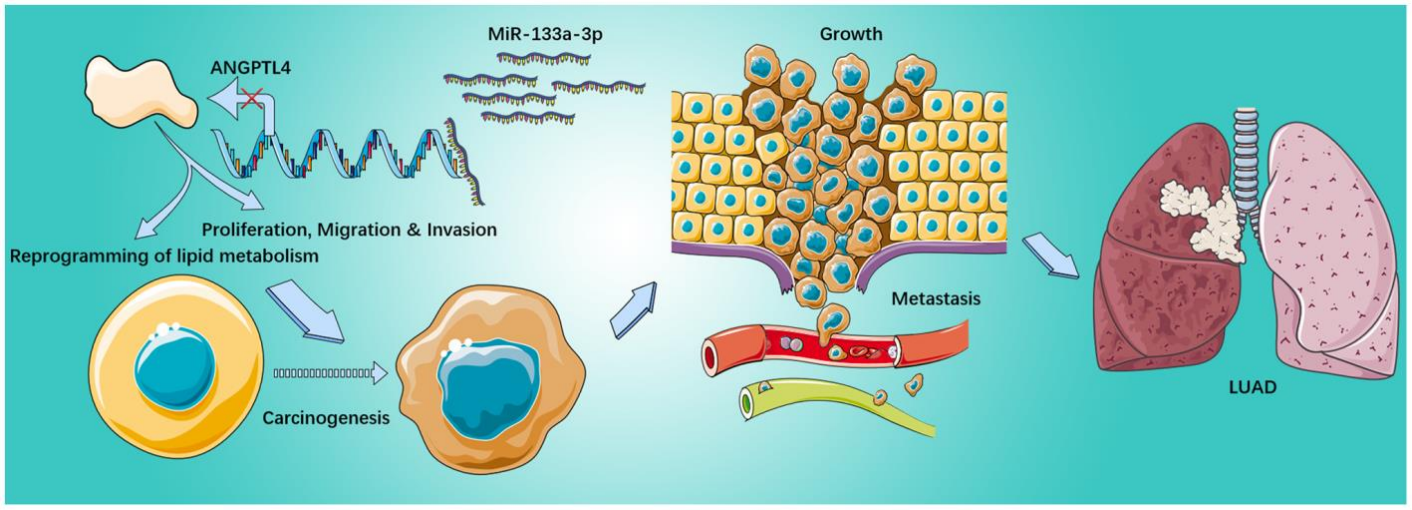
The potential mechanisms of miR-133a-3p/ANGPTL4 axis in LUAD.

In conclusion, we found ANGPTL4 was significantly increased in LUAD, and miR-133a-3p could directly repress ANGPTL4 expression and impeded LUAD proliferation, migration and invasion (Figure 6). Therefore, miR-133a-3p/ANGPTL4 axis might be a potential biomarker and therapeutic target for LUAD patients.

## Supplementary Materials

Supplementary Figure 1
