# Supplementary material for: ANGPTL4, a direct target of hsa-miR-133a-3p, accelerates lung adenocarcinoma lipid metabolism, proliferation and invasion
Source: Aging (Albany NY). 2023 Dec 29;16(9):8348–60. doi: 10.18632/aging.205313 (PMC11132016; doi:10.18632/aging.205313)
Supplement: Supplementary Figure 1 [file aging-16-205313-s001.pdf]

SUPPLEMENTARY FIGURE

	Predicted consequential pairing of target region (top) and miRNA (bottom)	Site type	Context++ score	Context++ score percentile	Weighted context++ score	Conserved branch length	P_{CT}	Predicted relative K_D
Position 285-291 of ANGPTL4 3' UTR<br>hsa-miR-133a-3p.1	5' ...AGUUGGGGACUCAGAGGGACCAC...<br>||||||| 3' GUCGACCAACUUCCCCUGGUU	7mer-m8	-0.39	95	-0.39	4.416	0.85	-4.676

Supplementary Figure 1. Predicted consequential pairing of target region of ANGPTL4 and miR-133a-3p.
